# River Metabolism along a Latitudinal Gradient across Japan and in a global scale

**DOI:** 10.1038/s41598-019-41427-3

**Published:** 2019-03-20

**Authors:** Anandeeta Gurung, Tomoya Iwata, Daisuke Nakano, Jotaro Urabe

**Affiliations:** 1 Graduate School of Life Sciences, Tohoku University, 6-3 Aoba, Aramaki, Aoba-ku, Sendai 980-8578 Japan; 2 Faculty of Life and Environmental Sciences, University of Yamanashi, 4–3–11 Takeda, Kofu, 400–8511 Japan; 3 River and Coastal Environmental Sector, Environmental Science Research Laboratory, Central Research Institute for Electric Power Industry (CRIEPI), 1646 Abiko, Chiba, 270 - 1194 Japan

## Abstract

Since temperature is a key factor affecting photosynthetic and respiration rates, the rates of gross primary production (GPP) and ecosystem respiration (ER) are expected to be lower for rivers at higher latitudes, while the net ecosystem production (NEP) rate likely decrease in rivers at lower latitude due to higher sensitivity of ER to temperature compared with GPP. To examine these possibilities, we estimated the ecosystem metabolism of 30 rivers located from 43.03°N to 32.38°N in Japan during summer using a Bayesian model with hourly changes in dissolved oxygen concentrations. In addition, we examined latitudinal trends of GPP, ER and NEP in a global scale by compiling and analyzing river metabolic data estimated in previous studies. Our analysis showed that both GPP and ER tended to increase with latitude, although these rates were positively related to water temperature in Japanese rivers. Global dataset of GPP and ER also showed increasing trend towards higher latitude. In addition, contrary to our initial expectations, NEP decreased with latitude and most rivers were net heterotrophic at both regional (Japanese rivers) and global scales. These results imply that the latitudinal temperature effect on river metabolism is masked by other factors not examined in this study, such as land use in the watershed, which play pivotal roles in explaining the latitudinal variation of river metabolism.

## Introduction

Ecosystem metabolism includes carbon fixation and mineralization through gross primary production (GPP) and ecosystem respiration (ER). In rivers, the balance between GPP and ER, denoted by net ecosystem productivity (NEP), is not necessarily positive since, in addition to organic carbon fixed by autotrophs within rivers, terrigenous organic carbon is discharged into rivers and respired^1,2^. This implies that river communities are sustained by both autochthonous and allochthonous organic carbon and that the community dependency on the terrigenous carbon is reflected by the balance of fixation and mineralization of organic carbon. Thus, GPP, ER and NEP are important properties integrating the biological processes of communities involved and characterizing given river ecosystems^2,3^.

River metabolism is known to be influenced by various abiotic and biotic factors such as light^3–8^, temperature^7,9,10^, nutrients^11–13^, hydromorphology^14,15^, geomorphology^16,17^, and changes spatially and seasonally. Among these, previous studies have shown that GPP is affected mainly by light^3–8^ and temperature^7,9,10^, while ER is controlled mainly by temperature^4,9,16,18,19^. In addition, some studies suggested that ER is more responsive to temperature compared with GPP^20,21^. Thus, GPP, ER and NEP in rivers may systematically change with the latitudinal gradient of light and temperature. If this were the case, the latitudinal gradient would be useful to build predictive models of river ecosystem metabolism in response to warming and climate changes. However, although a few study have examined the latitudinal variations of GPP^22^ in rivers, no study has yet examined latitudinal variations of ER and thus NEP.

In this study, therefore, we simultaneously estimated GPP, ER and NEP in rivers at various latitudes in Japan. Since Japan extends over a wide range of latitudes from 24°N to 45°N, it provides an excellent location to examine these hypothesis. In Japan, the Ministry of Land, Infrastructure, Transport and Tourism (MLIT) has routinely measured diurnal changes in DO and water temperature at monitoring sites of major rivers and provides these data as an open access database (Water Information System, http://www1.river.go.jp). Using these measured DO values with a modern statistical modelling, we estimated GPP, ER and NEP in various rivers of Japan. Then, we examined the following hypotheses: (1) GPP would decrease towards the north since both temperature and the amount of solar radiation decrease from lower to higher latitudes, (2) ER would also be lower in areas of higher latitude since lower temperatures reduce the respiration activity of organisms; and accordingly, and (3) river ecosystems would become more heterotrophic in rivers located at lower latitude since ER is more sensitive to changes in temperature than GPP^20,21^. Finally, to test if the latitudinal trends of GPP, ER and NEP found in Japan rivers are valid in a spatially larger scale, we complied and examined literature data on GPP and ER estimated in rivers at various latitude in the world and compared the latitudinal trends with those in Japanese rivers.

## Materials and Methods

### Study area

The Japanese Archipelago (area: 377,880 km^2^) extends over approximately 2,000 km from subtropical in the south to subarctic climatic conditions in the north^23,24^ and has four distinct seasons^25^. In general, summer extends from mid-June to September, with early summer experiencing a rainy season, known as the Tsuyu. During the late summer and autumn, typhoons strike the archipelago, which often result in heavy rains and river flooding. In the northern areas, snowfall occurs during the winter, when river flow is generally low. In such snow-covered areas, river flow becomes high during the spring with snowmelt runoff. As a result, river flow fluctuates seasonally and annually depending on the rainfall and snowmelt patterns^26^. Geologically, Japan is characterized by frequent tectonic and geothermal activity. Japanese rivers are generally short (max length: 370 km) and steep, with flashy flow regimes and thus are sediment rich^24^.

In this study, we focused on the metabolic rates in August since it falls before the typhoon season and after the early summer rainy season, and thus the weather conditions are relatively stable throughout the country. In addition, the high temperatures in this month cause high biological activity, which likely intensifies the latitudinal gradients.

### Data collection

We collected river data in August from the database constructed by the Water Information System (WIS: http://www1.river.go.jp/) developed by MLIT, except for Mimi River (ID = 30, Table 1), which was provided by the Central Research Institute of Electric Power Industry (CRIEPI). The WIS database provides water level, discharge, DO, pH, conductivity and water temperature data that have been measured hourly at 90 observatory river stations throughout Japan. Since these river stations were setup originally to monitor water flows and make a risk assessment of flood and water related disasters, the stations were located at the mid to down streams of the rivers and only a limited number of river stations had periodical measurements of DO concentrations. In addition, river stations were less located in the northern areas.

**Table 1:** Mean metabolic values and reaeration constants of rivers computed by the BASE model in this study.

ID	River	Observatory Site	Latitude	Longitude	Mean GPP (g C m^−2^ d^−1^)	Mean ER (g C m^−2^ d^−1^)	NEP (g C m^−2^ d^−1^)	Mean K (d^−1^)
1	Toyohiragawa	Horohirabashi	43.0377	141.3555	0.28	0.76	−0.47	32.80
2	Tokachigawa	Tokachibashi	42.9344	143.2033	0.17	0.04	0.13	0.30
3	Chitosegawa	Hinodebashi	42.8325	141.6597	0.50	2.16	−1.65	14.03
4	Iwakigawa	Goshogawara	40.8077	140.4375	1.89	4.30	−2.41	6.31
5	Iwakigawa	Kamiiwakibashi	40.5919	140.4169	3.17	3.98	−0.81	18.73
6	Kitakami	Funada bashi	39.8355	141.1613	2.38	1.42	0.96	25.82
7	Kitakami	Shiwabashi	39.5513	141.1755	2.29	2.64	−0.35	7.38
8	Kitakami	Kanegasaki hashi	39.1966	141.1272	3.82	3.73	0.09	5.58
9	Mogamigawa	Horinouchi	38.6641	140.2730	1.07	1.01	0.06	2.15
10	Shinanogawa	Shinanogawa	37.8816	139.0188	2.34	3.52	−1.18	1.94
11	Kujigawa	Sakakibashi	36.4963	140.5544	0.84	1.19	−0.36	7.13
12	Tonegawa	Ashikaga	36.3269	139.4530	0.77	1.20	−0.43	19.45
13	Kisogawa	Kasamatsu	35.3613	136.7569	4.70	5.75	−1.06	6.40
14	Yuragawa	Shimoamadzu	35.3555	135.1152	0.16	0.21	−0.05	3.75
15	Nagaragawa	Ōyabu ōhashi	35.2966	136.6711	0.05	0.06	−0.01	1.50
16	Shōnaigawa	Biwajima	35.1991	136.8747	2.63	2.83	−0.20	8.51
17	Yahagigawa	Iwatsu	35.0022	137.1666	1.55	1.50	0.05	6.58
18	Katsuragawa	Miya Maebashi	34.9075	135.7166	1.21	1.30	−0.09	5.95
19	Ujigawa	Miyukibashi	34.8911	135.6994	1.42	2.45	−1.03	4.33
20	Inagawa	Ginbashi	34.8555	135.4155	2.50	1.35	1.15	18.72
21	Yodogawa	Hirakata Ōhashi	34.8125	135.6316	1.51	2.12	−0.60	2.43
22	Toyokawa	Tō furu/Tougo	34.8105	137.4186	0.71	0.66	0.05	1.86
23	Ibogawa	Kamikawara	34.8013	134.5630	0.90	1.22	−0.32	5.51
24	Inagawa	Gunkōbashi	34.7988	135.4233	1.76	2.01	−0.25	10.39
25	Kakogawa	Kunikane	34.7975	134.8994	1.81	1.95	−0.14	1.97
26	Kumozugawa	Kumozubashi	34.6466	136.5130	0.45	0.34	0.10	1.36
27	Yamatogawa	Asaka	34.5858	135.5019	2.33	5.12	−2.78	11.55
28	Miyagawa	Watarai-bashi	34.4891	136.6855	0.54	0.52	0.02	2.64
29	Chikugogawa	Kurumeōhashi	33.3292	130.5261	1.64	1.51	0.13	3.41
30	Mimigawa	Yamagehei	32.3862	131.5264	2.99	1.61	1.39	5.04

In this study, we first downloaded dataset from the years 2010–2016 and determined whether continuous 24-hour time series data were available. Unfortunately, DO data were often temporally missing, deviated from their natural range relative to temperature (0–15 mg O_2_/L), drifted strongly in a short period or showed temporally unchanged values, probably due to troubles or malfunctions with DO sensors. Since there were no remarks about these troubles on DO sensors in the website, we removed days when DO showed these unusual values. Accordingly we used data at dates when DO concentrations showed distinct diel patterns of DO as in Fig. S1a,b.

**Figure 1:**
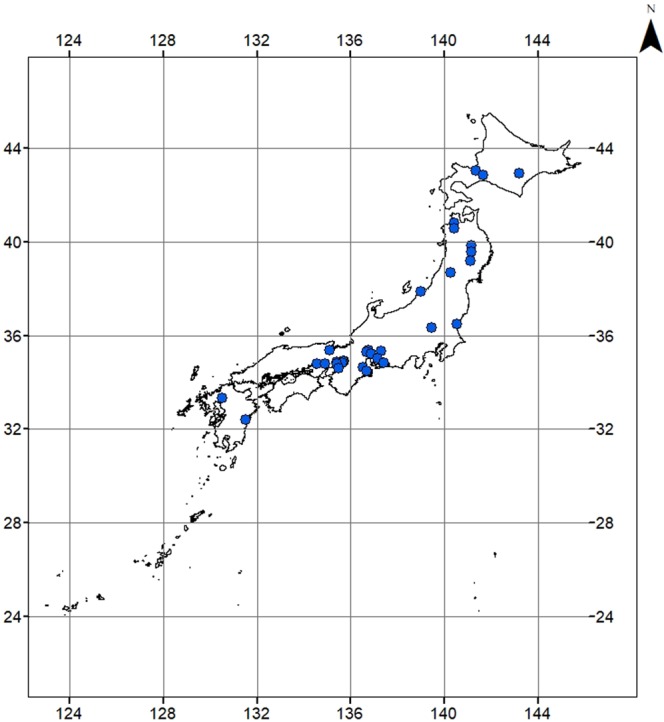
Map of Japan showing the river observatory stations where the dissolved oxygen (DO) and water temperature data were collected. Details of each river are shown in Supplementary Table S1.

We verified the data consistency by confirming availability for at least 3 days in August of each year from 2010 to 2016. Based on the availability (number of days) and reliability (if the values were within naturally reasonable range) of 24-hour time series data, 30 river stations from 43.03°N to 32.38°N were selected (Fig. 1, Table S1) with a total of 110 values from multiple years at these stations.

We obtained stream order at each river station from 10 m digital elevation maps provided by the Geospatial Information Authority of Japan (https://fgd.gsi.go.jp/download/menu.php) with the Spatial Analyst tool of ArcMap 10.5^27^. Since water depth was not recorded at the MLIT observatory river stations, we estimated mean depth (*D*) for each river using the discharge data (*Q*, m^3^/s) and Manning’s equation by assuming that all rivers had a rectangular cross section. Discharge data were obtained from the WIS database, while water surface slope and wetted width (*W*) were estimated remotely using the Add Path tool in Google Earth Pro. Manning’s roughness constant for natural channels was selected from Coon^28^ depending on the type of channel morphology. Then, the mean water depth *D* was estimated using following equations,

1 $$D=\frac{Q}{V\times W}$$

where *V* is mean water-column velocity (m/s) estimated by Manning’s equation as follows,

2 $$V=\frac{{H}^{\frac{2}{3}}\times {S}^{\frac{1}{2}}}{n}$$

where *H* is hydraulic radius, *S* is the water surface slope, and *n* is the channel roughness constant. We used this estimated depth rather than the depth at the pin-point location of the MLIT observatory river station since the metabolic rate measurements are not necessarily products of the river station alone but those of upstream area over 10–10^4^ m^29,30^.

Hourly data of meteorological parameters such as atmospheric temperature, pressure, precipitation, cloud cover and irradiance were obtained from the meteorological stations of the Japan Meteorological Agency (http://www.jma.go.jp/jma/index.html) that were closest to the river stations. Irradiance data collected at the meteorological stations were converted to photon flux using a conversion factor of 0.46^31^. According to the areal images, all the river stations had an open canopy.

### Model estimating the metabolic rates

Various models have been developed for estimating reaeration rate^17,32,33^ to estimate primary production and ecosystem respiration rates from daily DO profiles. Among these, we used the BASE v2.0 (Bayesian Single Station Estimation) model developed by Grace *et al*. (2015) to estimate GPP and ER because it was made publicly available and could easily compute large number of dataset in a short period of time. In addition, our dataset including DO, water temperature and irradiance met the requirement of the BASE model.

BASE v2.0 is a model based on the daytime regression developed by Kosinski^34^ which describes the DO concentration (mg O_2_/L) at time step *t* + 1 from the primary production, ecosystem respiration and reaeration rate at preceding time step *t* as follows:

3 $$\,{[DO]}_{t+1}={[DO]}_{t}+A{I}_{t}^{p}-R({\theta }^{({T}_{t}-\bar{T})})+K({1.0241}^{({T}_{t}-\bar{T})})\,{D}_{t}$$

where $A{I}_{t}^{p}$ refers to the volumetric primary production rate (mg O_2_ L^−1^ d^−1^), *A* is a constant value representing the primary production per quantum of light, *I* is the incident light intensity at the water surface (µmol m^−2^ s^−1^), *p* is an exponent reflecting the ability of primary producers to use incident light, *R* is the volumetric ecosystem respiration rate (mg O_2_ L^−1^ d^−1^), *θ* is the temperature dependent factor of the respiration rate, *T* is water temperature (°C), $\bar{T}$ is mean water temperature over the 24-h period, *K* (d^−1^) is the reaeration coefficient, and *D* is the difference between the measured DO concentration and the saturated DO concentration at a given temperature, salinity and barometric pressure.

By fitting the equation to recorded data, parameter values of production, respiration and reaeration rates (*A*, *p*, *R*, *θ, K*) were empirically obtained. This model was called from a script in the statistical software R, which involves JAGS to run the Markov Chain Monte Carlo (MCMC) iterations^33^. The program run was performed with the time interval set to 3600 (for one-hour interval) for 20,000 to 200,000 iterations. We excluded the daily data from the further analyses if no model convergence was obtained after the maximum MCMC iterations. We also removed dates that showed very poor model fit of O_2_ data even when the parameter chains converged.

The BASE v2.0 model provided the means and the standard deviations for the daily volumetric metabolic rates and the other estimable parameters (*A*, *p*, *R*, *θ, K*), as well as instantaneous rates of volumetric GPP and ER for each time step. The output for the diel model produced multi-panel validation plots that helped assess the convergence of the model. Quantitatively, these were assessed by checking the posterior predictive *p*-value, *R*^2^ value, and the residual mean square error values (RMSE). Validation plots included MCMC trace plots for the parameter values. Upon a successful convergence of the model, all five chains (*A*, *p*, *R*, *θ, K*) of parameters overlapped and became centred (Fig. S2).

### Collection of literature data

To compare river metabolic rates obtained in Japanese rivers with those in other regions, we collected GPP and ER, and calculated NEP (GPP – ER) in rivers at various latitudes from 27 previously published studies (Table S3) and examined if these rates varied along latitudes even at a spatially larger scale.

### Statistical analysis

In this study, we converted GPP and ER values into units of carbon, assuming both photosynthetic and respiration quotients of unity. We also converted the volumetric metabolic rates into areal estimates by multiplying by the mean water depth, which was determined from the discharge data and Manning’s equation. Before statistical analyses, we screened the data for outliers. Then, mean metabolic rates (GPP, ER and NEP) in August were calculated for each site for each year during the period from 2010 to 2016.

Since the elevation and PAR data were highly skewed, we log-transformed them before the analysis. To examine if the metabolic rates were related to latitude, we analysed these with Generalized Linear Mixed Model (GLMM) using latitude as a fixed factor and year as a random factor by using the *lmer* function of the lme4 package version 1.1^35^ of R version 3.3.2^36^. Relationship between metabolic rates and latitudes were then examined by simple regression analyses with data estimated in Japanese rivers and collected from literatures using R version 3.3.2^36^.

To examine the direct and indirect effects of latitude and other explanatory variables on the metabolic rates, we performed structural equation modelling (SEM) using data obtained in Japanese rivers and considering the causal relationships among the metabolic rates and the explanatory variables. In this analysis, we used latitude, elevation, water temperature and PAR as explanatory variables. We excluded stream order in SEM because simple correlation test showed no significant relationship with metabolic values. We standardized all the explanatory variables before the analysis. Within single rivers, we treated the monthly average of metabolic rates for a year as an independent data. Thus, we examined total 110 values for each of GPP, ER and NEP. In SEM, model fitting was performed using maximum-likelihood estimation, and the relative importance of each path was compared using individual path coefficients. A chi-square test was used to quantify the overall fit of the model. SEM was performed using the *lavaan* package version 0.5^37^ of R version 3.3.2.

## Results

**Figure 2:**
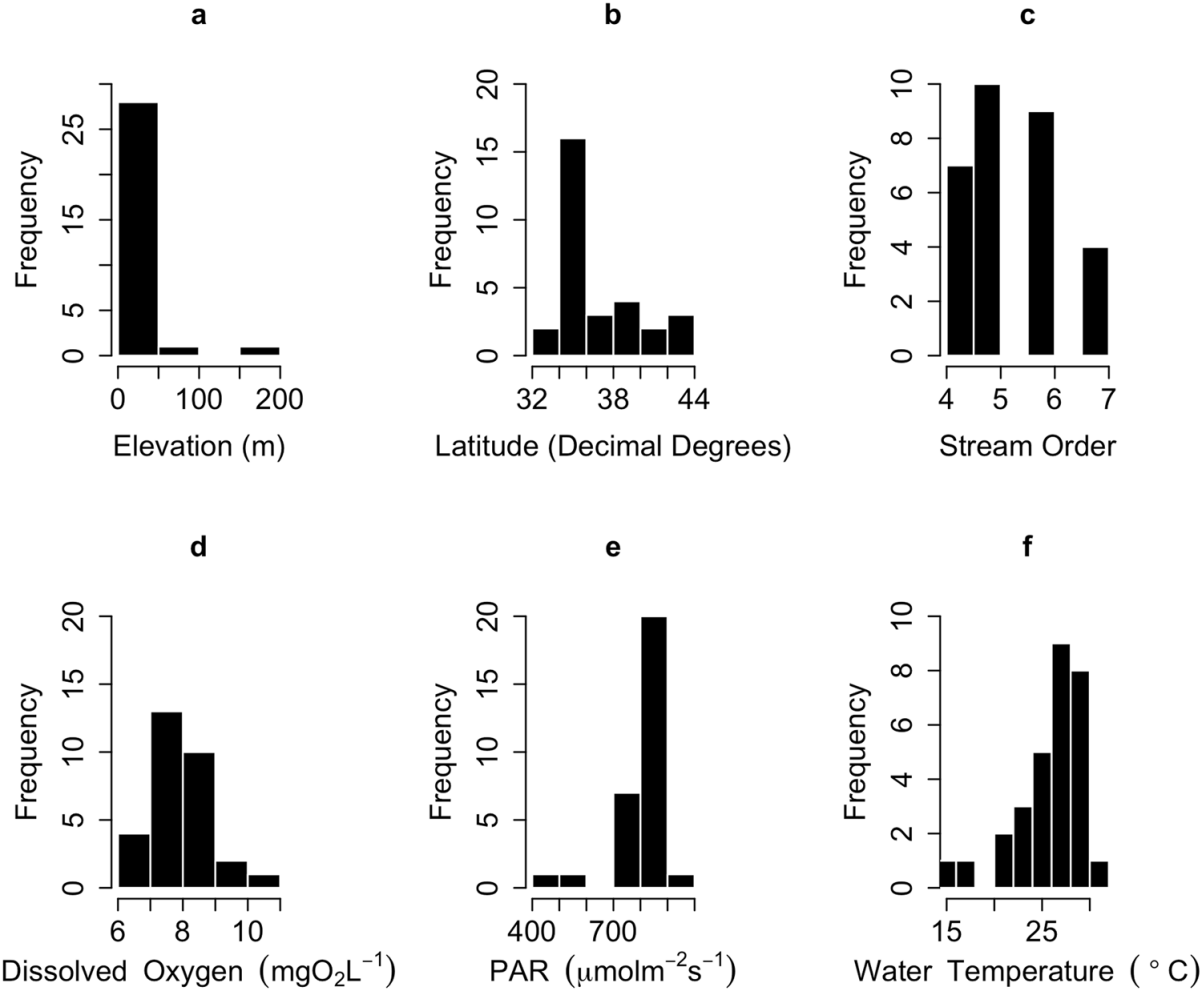
Frequency histogram of independent parameters used in the study. (**a**) Elevation, (**b**) Latitude, (**c**): Stream Order, (**d**): Dissolved Oxygen (DO), (**e**): Photosynthetically Active Radiation (PAR) and (**f**) Water temperature.

**Figure 3:**
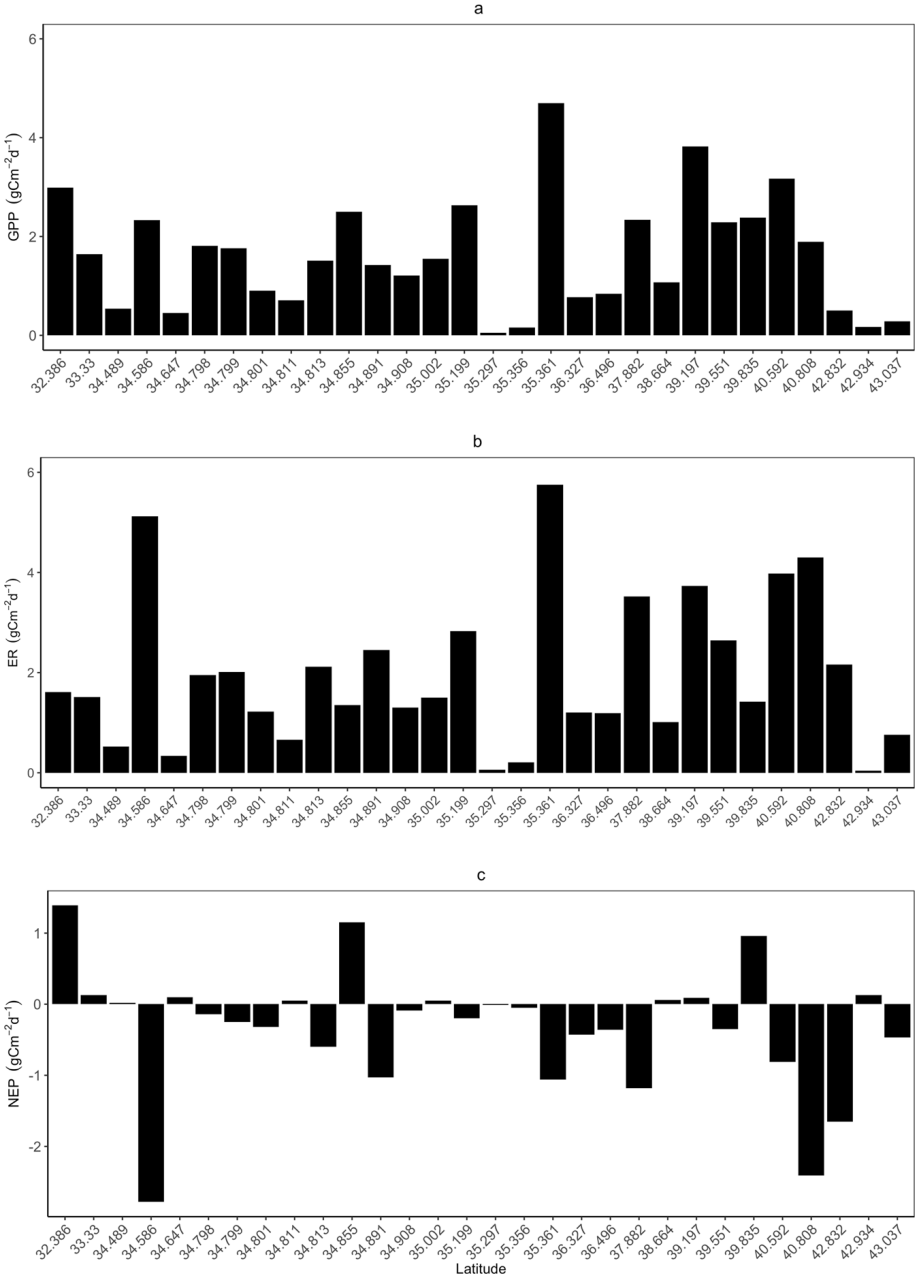
River metabolic rates along the latitudinal gradient in Japan. The x-axis shows latitude (from the south to the north), and the y-axis shows GPP (upper panel), ER (mid panel) and NEP (lower panel) in units of carbon. Individual metabolic rates for each river are shown in Table 1.

The river observatory stations used in this study sprawled across the entire archipelago from Hokkaido to Kyushu (Fig. 1; Table 1). The elevation of the observatory stations ranged from 1 m at the Shinano River to 181 m at the Kitakami River (Fig. 2; Table S1). The rivers examined were mid to large sized, with stream orders ranging from 4 to 7 (Table S1). Mean DO concentration in August ranged from 4.90 to 10.58 mg O_2_/L and that of PAR ranged from 457 to 1062 μmol m^−2^ s^−1^ (Fig. 3; Table S1). Mean water temperature in August ranged from 15.3 °C at the Toyohira River to 31.9 °C at the Yodo River.

**Figure 4:**
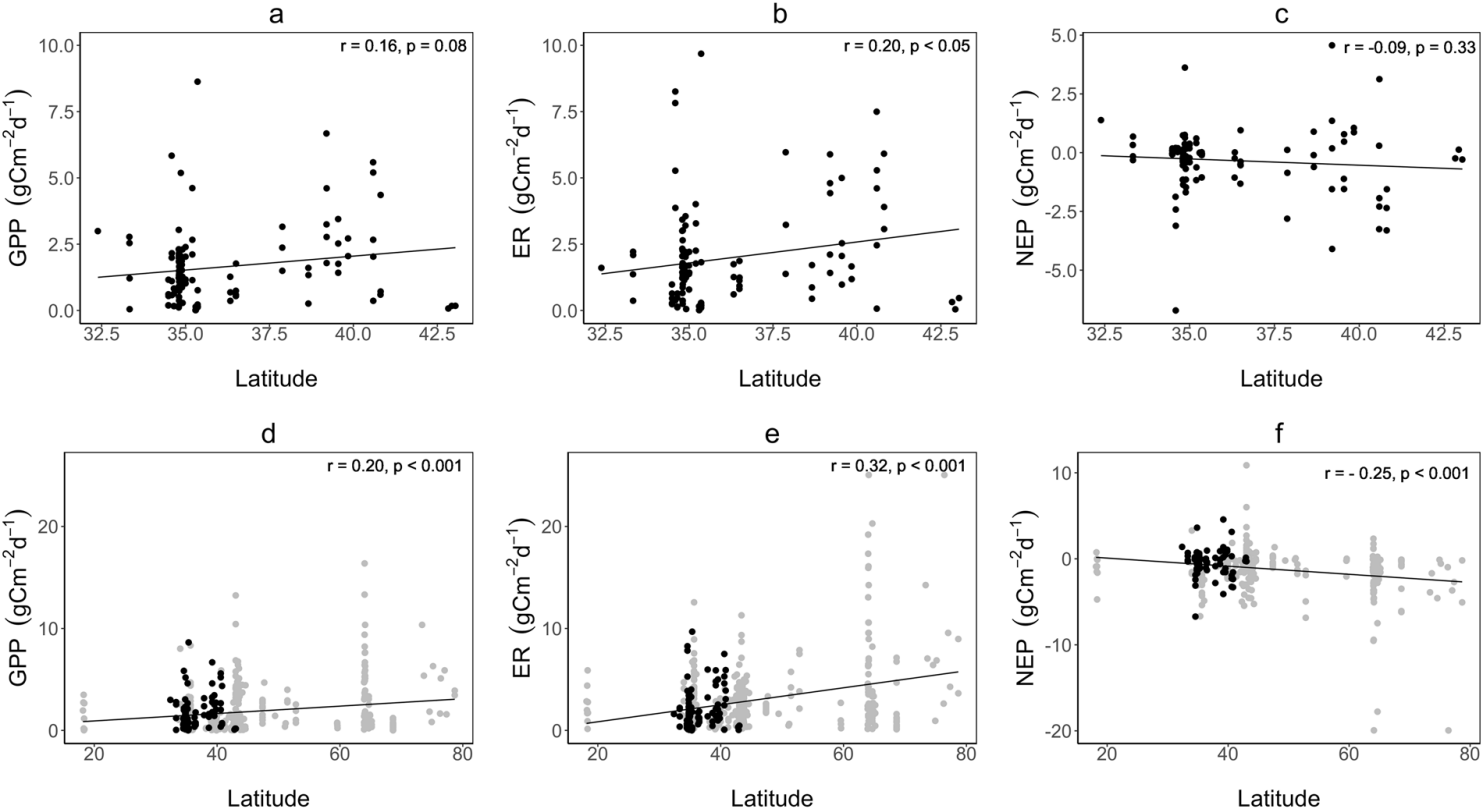
Gross primary production rate (GPP), ecosystem respiration rate (ER) and net ecosystem productivity (NEP) of the rivers in Japan (**a**–**c**: regional scale) and various areas (**d**–**f**: global scale) plotted against latitude. The results of GLMM with the statistical values are shown in Supplementary Table S3.

We obtained a total of 646 estimates for each of gross primary production (GPP) and ecosystem respiration (ER), with 110 mean values for August in different years and at different river stations (3–15 data points per river station). Reaeration rates (K) ranged from 0.01 day^−1^ to 33.87 day^−1^, with a mean of 7.33 day^−1^. GPP varied highly, with the estimates ranging from 0.01 g C m^−2^ d^−1^ at the Nagara River (ID = 15, n = 110) to 8.62 g C m^−2^ d^−1^ at the Kiso River (ID = 13, n = 110). Similarly, ER ranged from 0.01 g C m^−2^ d^−1^ at the Nagara River to 9.68 g C m^−2^ d^−1^ at the Kiso River. Model estimate of K was significantly and positively correlated with both GPP (r = 0.32, p < 0.005) and ER (r = 0.26, p < 0.005) (Fig. S4). Across all river stations, GPP covaried positively and significantly with ER, although a few rivers, such as the Iwaki River (ID = 5) and the Yamato River (ID = 27), had higher ER values without correspondingly high GPP values (Figs 3, S3). On average, NEP ranged from −2.78 g C m^−2^ d^−1^ at the Yamato River to 1.39 g C m^−2^ d^−1^ at the Mimi River (ID = 30), and only 11 out of 30 rivers had positive NEP (Table S1; Fig. S). GLMM showed that latitude affected significantly GPP and ER but not NEP (Table S2). In simple regression analyses, GPP was marginally and ER was significantly higher in rivers at higher latitude (Fig. 4a,b), while no significant relationship was detected between NEP and latitude (Fig. 4c).

To test the generalities of the latitudinal trends found in Japanese rivers, we complied and examined GPP and ER in rivers located from 18°N to ~78°N that were estimated in 27 previous studies (Table S3). Both GPP and ER from literature and in this study ranged from 0 to 20 g C m^−2^ d^−1^. In addition, the literature data showed that GPP and ER increased significantly towards higher latitude in accord with the trends found in Japanese rivers (Fig. 4d,e). Similar to Japanese rivers, most of the NEP from literature showed negative values. In the case of data from literature, NEP tended to show significantly lower values at the higher latitude (Fig. 4f).

**Figure 5:**
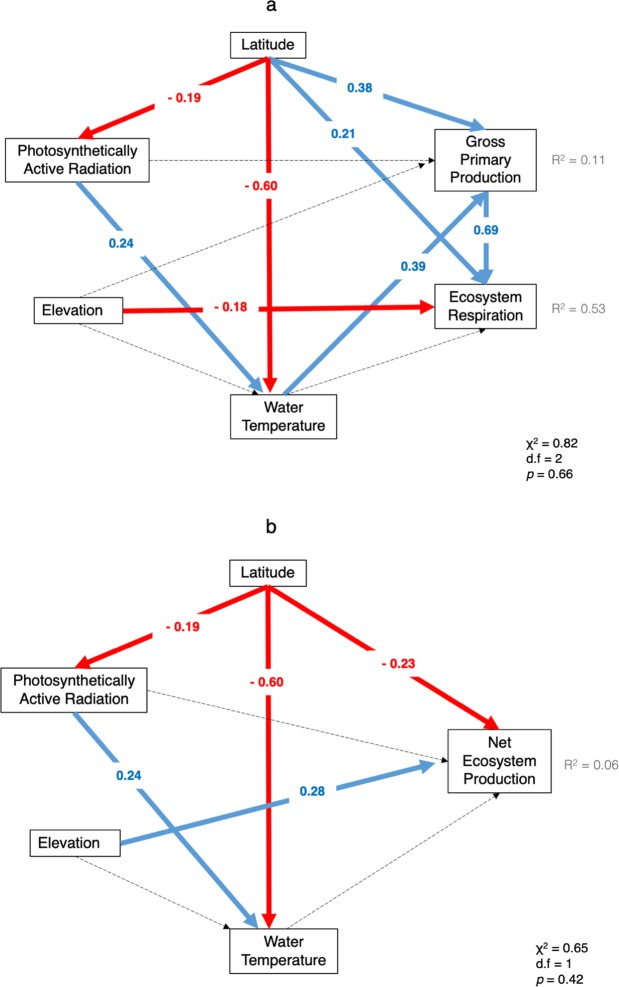
Results of the structural equation model, showing direct and the indirect effects of latitude and other factors on gross primary production rate (GPP), ecosystem respiration rate (ER) and net ecosystem production rate (NEP) of the rivers in Japan. Strengths of effects are denoted by path coefficients (i.e., regression coefficients). Red and blue lines indicate significantly negative and positive paths (p < 0.05), respectively, and dashed lines indicate hypothesized pathways that were not significant in the model. The amount of variation explained by the model is given by R^2^ with fit statistics in each panel. Elevation and PAR are log transformed.

Structural equation modelling (SEM) explained 11% and 53% of variations in GPP and ER in Japanese rivers with significant direct and indirect effects of explanatory variables on these metabolic rates (Fig. 5a). In the model, latitude had a significant positive direct effect on both GPP (standardized effect = 0.38) and ER (standardized effect = 0.21). Moreover, latitude had a significant indirect effect through negative effects of water temperature on GPP (standardized effect = −0.60 × 0.39 = −0.23). Although ER was not directly affected by water temperature, it was positively related to GPP (standardized effect = 0.69) and negatively related to elevation (standardized effect = −0.18).

PAR also showed a significant positive indirect effect on both GPP (standardized effect = 0.24 × 0.39 = 0.09) through water temperature. Latitude had a significant and negative direct effect on NEP (standardized effect = −0.23). However, no significant effects of water temperature were found for NEP. Instead, NEP was positively related to elevation (standardized effect = 0.28).

## Discussion

Since both photosynthesis and respiration rates in river ecosystems often depend on water temperature^4,7,9,10,16,19,38^, we first hypothesized that both gross primary productivity (GPP) and ecosystem respiration (ER) would systematically change with latitude. However, as opposed to our hypothesis, both GPP and ER increased along the latitude. To our knowledge, only one previous study^22^ examined the relationship between GPP and latitude for rivers between 30°N and 50°N, which failed to find any significant latitudinal effects. Dataset from 27 previous studies that examined rivers between 18°N and 78°N also showed increase in the GPP and ER at higher latitude. Therefore, increasing trends of river GPP and ER towards the north seems to be limited not only in Japan but occurs on much larger scales. In this study, water temperatures had positive effects on GPP in Japanese rivers, which in turn positively affected on ER.

Thus, although latitude can indirectly affect both GPP and ER through its negative effect on water temperature, other factors related with latitudinal gradients override this indirect effect. Other than temperature and irradiance, vegetation type, biomass, and anthropogenic land uses are known to change along latitude^39–41^. Thus, positive trends of GPP and ER along latitude may be caused by land use and land covers in the river watershed.

Estimation of reaeration rate in streams is important to properly estimate the ecosystem metabolism^3^. In this study, the reaeration rate was estimated by the BASE v2.0 model using the time series of DO data^33,42,43^. BASE v2.0 is certainly advantageous for estimating reaeration rate and thus stream metabolic rates with the statistical reliability. In general, reaeration rates estimated by model equations tend to underestimate compared with those estimated directly by gas tracer methods and empirical equations^3,44^. However, the degree of underestimation by the model equations was found to be small when the reaeration rate was less than 50 day^−1^ ^44^ and when the stream was deeper than 6 cm^3^. In this study, river depth was greater than 6 cm, and the estimated reaeration rate ranged from 0.01 day^−1^ to 33.87 day^−1^, with a mean of 7.33 day^−1^, within the range of values reported in previous studies^44,45^. Because we used a DO dataset measured at hourly intervals, our estimates of reaeration rates and metabolic rates might be sensitive to the precision of the data points. To increase the accuracy of the estimation of GPP and ER, we used the mean of the daily estimates of metabolic rates for at least three days during August as a single data point. The estimates of gross primary production rate (0.01 to 8.62 g C m^−2^ d^−1^) and ER (0.01 to 9.68 g C m^−2^ d^−1^) in this study are within the range of previous studies, suggesting that the river metabolic rates estimated in this study are reasonable and have not deviated greatly from the true values.

A significant indirect effect of PAR on GPP and ER through water temperature was seen in the SEM (Fig. 5). This presence of an indirect effect of PAR along with the absence of a direct effect of PAR on metabolic values suggest that the effect of light on the bottom of the river could have been camouflaged by other local factors such as turbidity and cloud cover. In general, the penetration of light to the bottom of a river decreases moving downstream, if all else is equal, because of the increasing water depth. In addition, with increasing stream order, rivers tend to receive more suspended particles and organic matter that increase the attenuation coefficient of light. In this study, we used PAR at the river surface and did not consider the attenuation coefficient of light in the river water^46,47^. Thus, the actual light level received by the autotrophs in rivers may have been not proportional to PAR at the surface.

Summer precipitation is generally lower in the northern areas compared to the southern areas of Japan^25^. Since inflows of nutrients and organic matter into rivers are expected to be higher in areas with greater precipitation, GPP and ER would be expected to be greater towards the south. However, as shown above, such latitudinal gradients in the metabolic rates were not found. The vegetation types also differ between northern and southern Japan^48^. For example, broad-leaved evergreen trees dominate the southern region, whereas coniferous trees and broad-leaved deciduous trees are predominant in the northern region. In addition, central and southern Japan are more urbanized and sustain greater population density than in the north^49^. Such latitudinal differences in land use and land cover may have directly or indirectly confounded the latitudinal trends in GPP and ER. Finer spatial analysis on the watershed would be essential to uncover the actual mechanisms of the effects of land cover and land use on river metabolism.

Among the 30 rivers examined in Japan, only 11 rivers showed positive NEP, indicating that most of these rivers are net heterotrophic in the summer, as has been reported in various rivers in other continents^5,7,10,15,50,51^. In this study, we hypothesized that river ecosystems are more heterotrophic in rivers located at lower latitude since ER is more sensitive to changes in temperature than GPP^20,21^. However, opposite to the hypothesis, both in regional (Japanese rivers) and global scales, NEP showed lower values in rivers at higher latitudes. Since this spatial trend cannot be explained by temperature, it may reflect higher allochthonous input relative to primary production in northern rivers. Our study also showed that elevation had a significant positive direct effect on NEP, with rivers at lower elevations exhibiting lower NEP than rivers further above sea level. These results suggest that the ecosystem respiration rate relative to primary production rate increased moving downstream, where more allochthonous organic matter from the upstream areas or the surrounding watersheds tends to accumulate. Previous studies^12,52^ also showed that downstream export of greater amounts of organic matter fuels heterotrophic respiration in rivers.

This study explained at most 11% and 53% of the spatial variation in summer GPP and ER of rivers in Japan through the direct and indirect effects of latitude, PAR and water temperature and elevation, indicating factors other than geographic position play pivotal roles in determining river metabolism. The development of epilithic algal biomass on riverbeds is a crucial determinant of GPP and depends highly on temporal variability in the flow rate of river waters^29^. The supply of nutrients associated with watershed anthropogenic activities also influences the algal biomass in rivers^53,54^. ER in rivers is also affected by the allochthonous supply of organic matter from agricultural and urban areas and riparian forests^1^. Thus, local environmental factors specific to individual rivers, which are related or unrelated to latitudinal gradients, may have masked the effects of the thermal gradient on GPP and ER.

In conclusion, although GPP and ER increased with increasing river water temperature, our analysis showed increase in GPP and ER and decrease in NEP toward higher latitudes, indicating that effects of latitude are not limited to temperature and are likely to include indirect effects of local environmental conditions. We first expected that a comparison of river metabolism along latitudinal gradients may be useful to predict the effects of putative warming on river ecosystems. However, latitudinal trends of GPP, ER and NEP found in this study suggest that the uniqueness of each river in conjunction with the latitudinally related factors such as land use and land cover confound the effects of temperatures on these metabolic rates. Thus, to better understand the effects of warming on river ecosystems, we should consider both local and latitudinal environmental conditions including vegetation types and biomass, and anthropogenic activities in the watershed.

## Supplementary information

Supplementary Information

## Data Availability

Available data will be uploaded to the Dryad Digital Repository upon acceptance.

## References

[1] Marcarelli AM, Baxter CV, Mineau MM, Hall RO (2011). Quantity and quality: Unifying food web and ecosystem perspectives on the role of resource subsidies in freshwaters. Ecology.

[2] Hotchkiss ER (2015). Sources of and processes controlling CO2 emissions change with the size of streams and rivers. Nat. Geosci..

[3] Mulholland PJ (2001). Inter-biome comparison of factors controlling stream metabolism. Freshw. Biol..

[4] Bott, T. L. *et al*. Benthic Comminity Metabolism Along The River Continuum. *Hydrobiologia* (1985).

[5] Roberts BJ, Mulholland PJ, Hill WR (2007). Multiple scales of temporal variability in ecosystem metabolism rates: Results from 2 years of continuous monitoring in a forested headwater stream. Ecosystems.

[6] Finlay JC (2011). Stream size and human influences on ecosystem production in river networks. Ecosphere.

[7] Beaulieu JJ, Arango CP, Balz DA, Shuster WD (2013). Continuous monitoring reveals multiple controls on ecosystem metabolism in a suburban stream. Freshw. Biol..

[8] Naiman RJ (1983). The annual pattern and spatial distribution of aquatic oxygen metabolism in boreal forest watershed. Ecol. Monogr..

[9] Demars BOL (2011). Temperature and the metabolic balance of streams. Freshw. Biol..

[10] Escoffier, N. *et al*. Estimating ecosystem metabolism from continuous multi-sensor measurements in the Seine River. *Environ. Sci. Pollut. Res*. 1–17, 10.1007/s11356-016-7096-0 (2016). 10.1007/s11356-016-7096-0 27335018

[11] Acuña V, Giorgi A, Muñoz I, Sabater F, Sabater S (2007). Meteorological and riparian influences on organic matter dynamics in a forested Mediterranean stream. J. North Am. Benthol. Soc..

[12] Iwata T (2013). Fluvial transport of carbon along the river-to-ocean continuum and its potential impacts on a brackish water food web in the Iwaki River watershed, northern Japan. Ecol. Res..

[13] Masese FO, Salcedo-Borda JS, Gettel GM, Irvine K, McClain ME (2017). Influence of catchment land use and seasonality on dissolved organic matter composition and ecosystem metabolism in headwater streams of a Kenyan river. Biogeochemistry.

[14] Young RG, Matthaei CD, Townsend CR (2008). Organic matter breakdown and ecosystem metabolism: functional indicators for assessing river ecosystem health. J. North Am. Benthol. Soc..

[15] Kupilas B, Hering D, Lorenz A, Knuth C, Gucker B (2017). Hydromorphological restoration stimulates river ecosystem metabolism. Biogeosciences.

[16] Uehlinger U (2006). Annual cycle and inter-annual variability of gross primary production and ecosystem respiration in a floodprone river during a 15-year period. Freshw. Biol..

[17] Atkinson BL, Grace MR, Hart BT, Vanderkruk KEN (2008). Sediment instability affects the rate and location of primary production and respiration in a sand-bed stream. J. North Am. Benthol. Soc..

[18] Webster, J. R., Wallace, J. B. & Benfield, E. F. Organic processes in streams of the eastern United States. *River stream Ecosyst. world* 117–187, citeulike-article-id:6945780 (1995).

[19] Acuña V, Giorgi A, Muñoz I, Uehlinger U, Sabater S (2004). Flow extremes and benthic organic matter shape the metabolism of a headwater Mediterranean stream. Freshw. Biol..

[20] Allen AP, Gillooly JF, Brown JH (2005). Linking the global carbon cycle to individual metabolism. Funct. Ecol..

[21] Song, C. *et al*. Continental-scale decrease in net primary productivity in streams due to climate warming. *Nat. Geosci*. **11** (2018).

[22] Lamberti GA, Steinman AD (1997). A comparison of primary production in stream ecosystems. J. North Am. Benthol. Soc..

[23] Iyama, S. Profile of Japanese Rivers - Background to River Engineering in Japan. *J. Hydrosci. Hydraul. Eng*. 1–4 (1993).

[24] Yoshimura C, Omura T, Furumai H, Tockner K (2005). Present state of rivers and streams in Japan. River Res. Appl..

[25] Japan Meteorological Agency. *Climate Change Monitoring Report 2015*. (2016).

[26] Yanai, S. In *Ecology of Riparian Forests in Japan* (eds Sakio, Hitoshi; Tamura, T.) 31–45 (2008).

[27] ESRI. Environmental Systems Research Institute (2017).

[28] Coon, W. F. *Estimation of roughness coefficients for natural stream channels with vegetated banks*. *U.S. Geological Survey water-supply paper* (1997).

[29] Allan, J. D. & Castillo, M. M. *Stream Ecology: Structure and Function of Running waters* (2007).

[30] Grace M, Imberger S (2006). Stream Metabolism: Performing & Interpreting Measurements. New South Wales Dep. Environ. Conserv. Stream Metab. Work. May.

[31] Wetzel, R. G. & Likens, G. *Limonological Analyses* (Springer US, 2000).

[32] Holtgrieve GW, Schindler DE, Branch TA, A’mar ZT (2010). Simultaneous quantification of aquatic ecosystem metabolism and reaeration using a Bayesian statistical model of oxygen dynamics. Limnol. Oceanogr..

[33] Grace MR (2015). Fast processing of diel oxygen curves: Estimating stream metabolism with base (BAyesian single-station estimation). Limnol. Oceanogr. Methods.

[34] Kosinski, R. J. A comparision of the accuracy and precision of several open-water oxygen productivity techniques (1984).

[35] Bates D, Maechler M, Bolker B, Walker S (2015). Fitting Linear Mixed-Effects Models Using {lme4}. J. Stat. Softw..

[36] R Core Team. R: A Language and Environment for Statistical Computing (2014).

[37] Rosseel Y (2012). {{lavaan}: An {R} Package for Structural Equation Modeling}. J. Stat. Softw..

[38] Hunt RJ, Jardine TD, Hamilton SK, Bunn SE (2012). Temporal and spatial variation in ecosystem metabolism and food web carbon transfer in a wet-dry tropical river. Freshw. Biol..

[39] Clavero, M., Villero, D. & Brotons, L. Climate change or land use dynamics: Do we know what climate change indicators indicate? *PLoS One* **6** (2011). 10.1371/journal.pone.0018581 PMC3080866 21533025

[40] Dixon RK (1994). Carbon pools and flux of global forest ecosystems. Science (80-.)..

[41] Dong J, Tucker C, Buermann W, Kaufmann R, Hughs M (2003). USDA Forest Service/UNL Faculty Remote Sensing Estimates of Boreal and Temperate Forest Woody Biomass: Carbon Pools, Sources, and Sinks carbon pools, sources, and sinks. Remote Sens. Environ..

[42] Saltarelli, W. A. *et al*. Variation of stream metabolism along a tropical environmental gradient. *J. Limnol*., 10.4081/jlimnol.2018 (2018).

[43] Dodds WK (2018). Spatial heterogeneity and controls of ecosystem metabolism in a Great Plains river network. Hydrobiologia.

[44] Young RG, Huryn AD (1999). Effects of Land Use on Stream Metabolism and Organic Matter Turnover. Ecol. Appl..

[45] Griffiths NA (2013). Agricultural land use alters the seasonality and magnitude of stream metabolism. Limnol. Oceanogr..

[46] Krause-Jensen D, Sand-Jensen K (1998). Light attenuation and photosynthesis of aquatic plant communities. Limnol. Oceanogr..

[47] Brandão LPM, Brighenti LS, Staehr PA, Barbosa FAR, Bezerra-Neto JF (2017). Partitioning of the diffuse attenuation coefficient for photosynthetically available irradiance in a deep dendritic tropical lake. An. Acad. Bras. Cienc..

[48] Numata M, Miyawaki A, Itow D (1972). Natural and semi-natural vegetation in Japan. Blumea.

[49] UNDESA. World Urbanization Prospects: The 2011 Revision. *Present. Cent. Strateg. …* 318, 10.2307/2808041 (2012).

[50] Hall RO, Tank JL, Baker MA, Rosi-Marshall EJ, Hotchkiss ER (2016). Metabolism, Gas Exchange, and Carbon Spiraling in Rivers. Ecosystems.

[51] Rovelli L (2017). Reach-scale river metabolism across contrasting sub-catchment geologies: Effect of light and hydrology. Limnol. Oceanogr..

[52] Cole JJ, Caraco NF (2001). Carbon in catchments: Connecting terrestrial carbon losses with aquatic metabolism. Mar. Freshw. Res..

[53] Miura, A. & Urabe, J. Changes in epilithic fungal communities under different light conditions in a river: A field experimental study. *Limnol. Oceanogr* (2017).

[54] Castillo MM (2010). Land use and topography as predictors of nutrient levels in a tropical catchment. Limnologica.

