# Supplementary material for: River Metabolism along a Latitudinal Gradient across Japan and in a global scale
Source: Sci Rep. 2019 Mar 20;9:4932. doi: 10.1038/s41598-019-41427-3 (PMC6426971; doi:10.1038/s41598-019-41427-3)
Supplement: Supplementary file 1 — Supplementary Information [file 41598_2019_41427_MOESM1_ESM.pdf]

Supplementary Information

River Metabolism along a Latitudinal Gradient across Japan and in a global scale

Anandeeta Gurung[1*], Tomoya Iwata[2], Daisuke Nakano[3] and Jotaro Urabe[1],

[1]Graduate School of Life Sciences, Tohoku University, 6-3 Aoba, Aramaki, Aoba-ku, Sendai, 980-8578, Japan

[2]Faculty of Life and Environmental Sciences, University of Yamanashi, 4–3–11 Takeda, Kofu 400–8511, Japan

[3]River and Coastal Environmental Sector, Environmental Science Research Laboratory, Central Research Institute for Electric Power Industry (CRIEPI), 1646 Abiko, Chiba 270 - 1194, Japan

*Corresponding Author: Anandeeta Gurung (gurung.anan@gmail.com)

Figure S1

Figure S2

Figure S3

Figure S4

Table S1

Table S2

Table S3

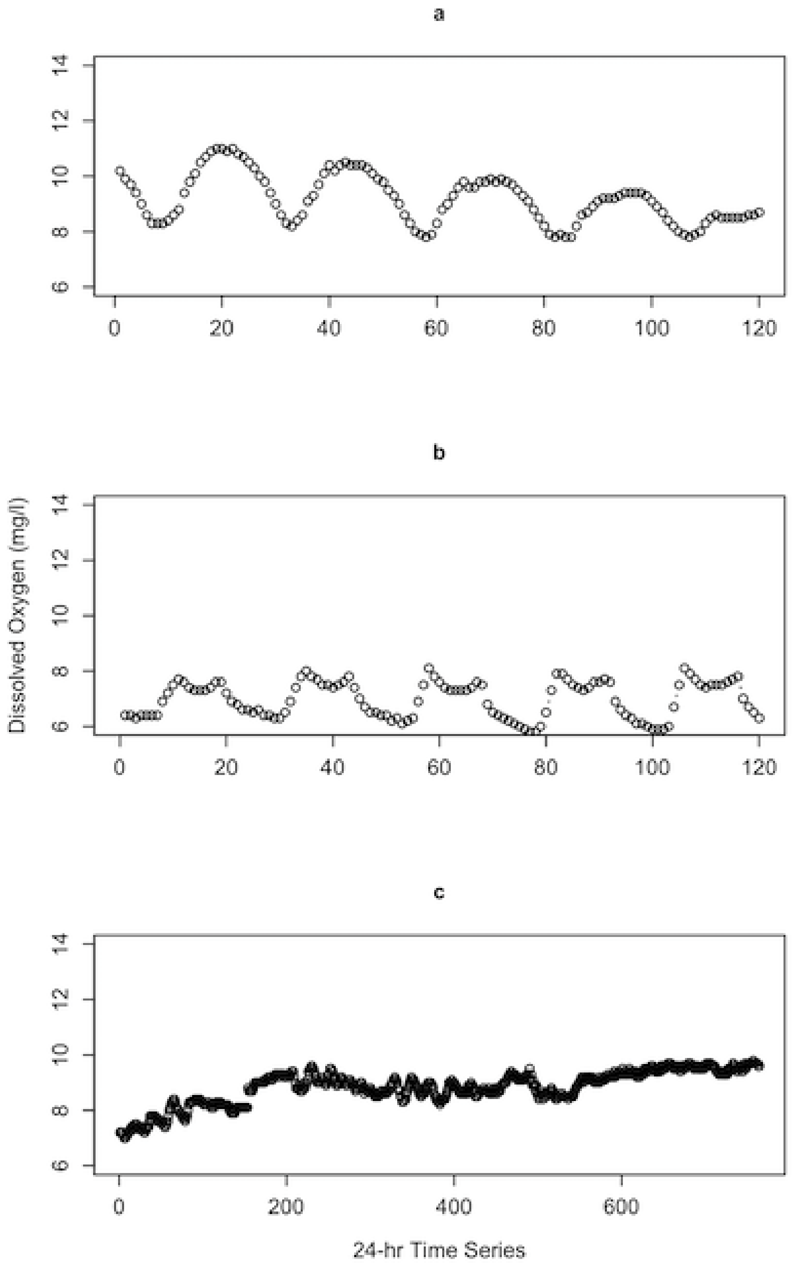

Fig. S1. Examples of patterns of diel dissolved oxygen (DO) data. (a) and (b) show examples of the distinct diel patterns that were included in the study, whereas erratic and inconsistent sites such as (c) were excluded. (a) Chikuko River, August 2012; (b) Shonai River, August 2016; and (c) Kitakami River, August 2015.

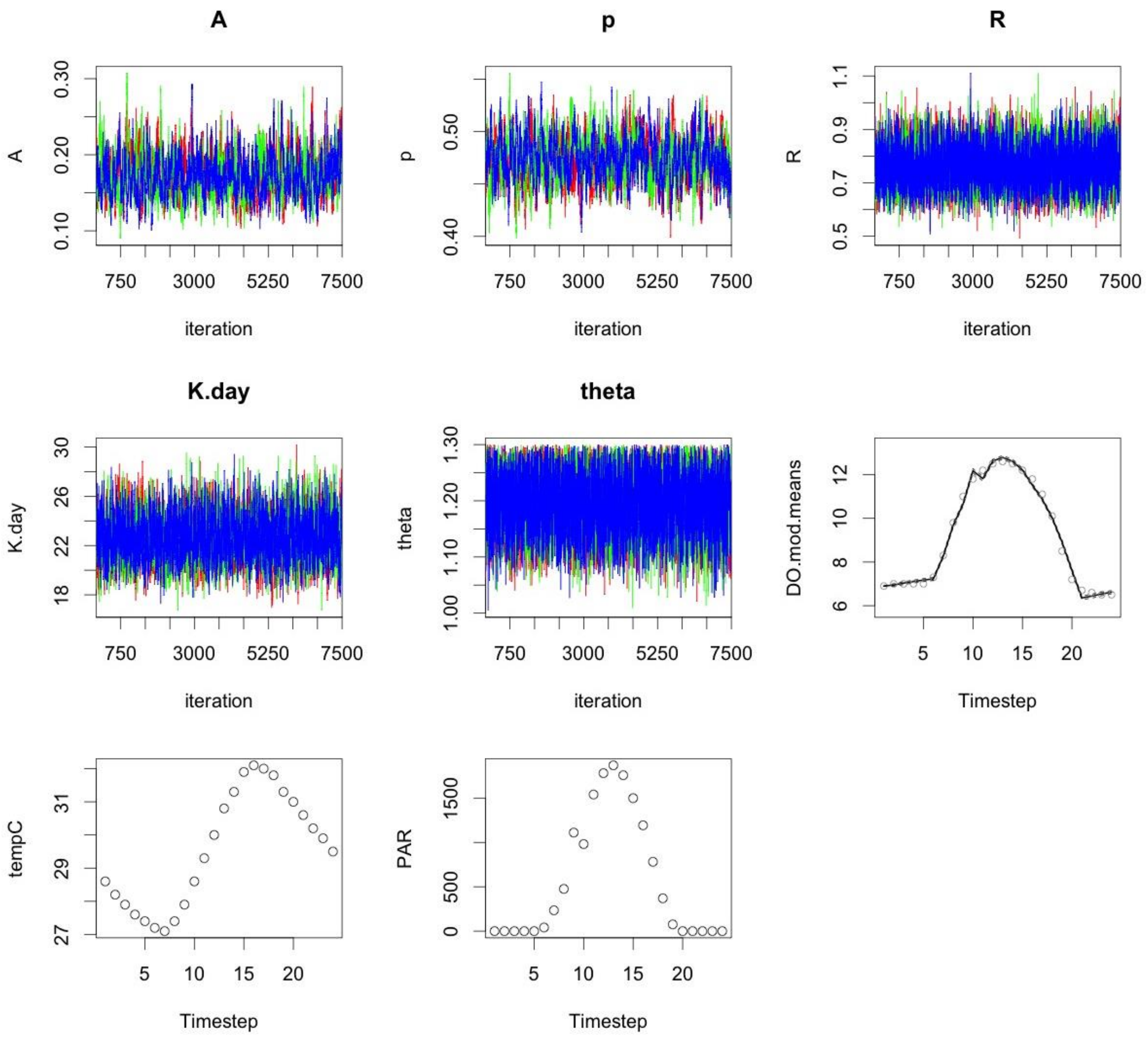

Fig S2. Example of validation plots for Ginbashi on the Ina River in Aug 4, 2015, obtained after running the model. Upon a successful convergence of the model, all five chains (A, p, R, K.day and theta) overlap and become centred. Plots of measured dissolved oxygen (DO) (empty circle) and predicted DO (black line), and measured temperature and photosynthetically active radiation (PAR) data are shown for each diel period.

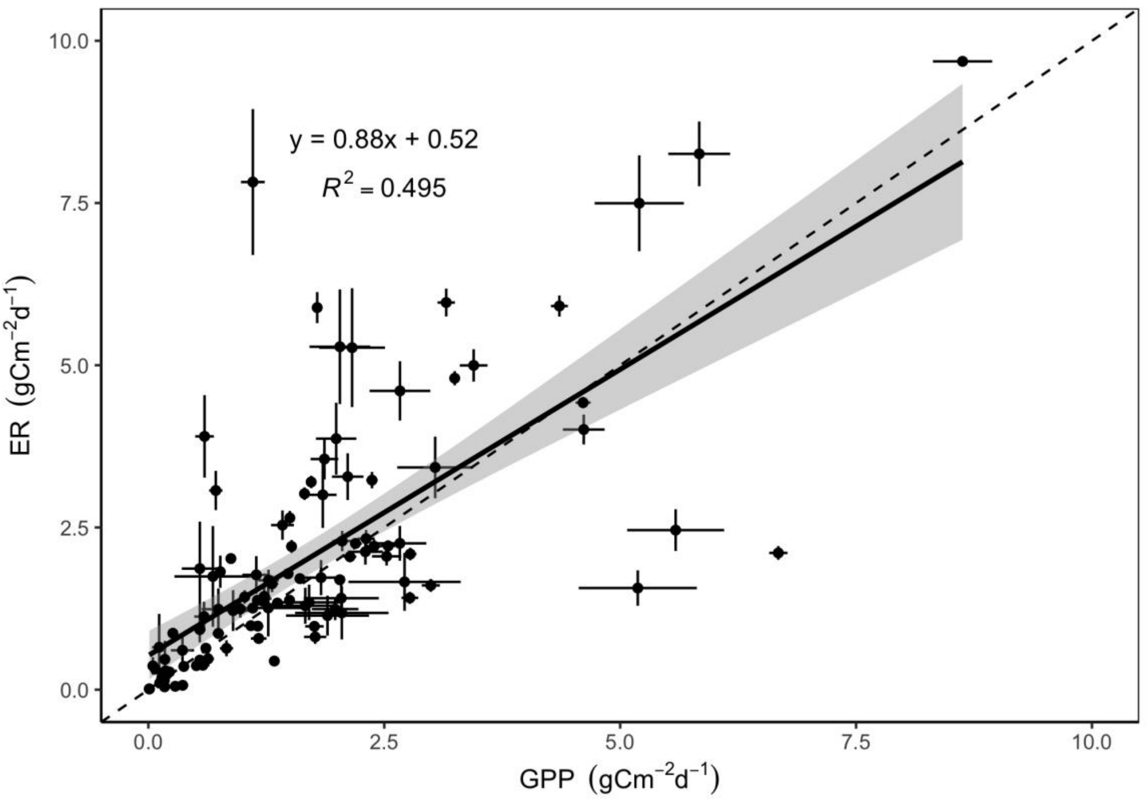

Fig. S3. Relationship between gross primary production rate (GPP) and ecosystem respiration rate (ER) across the rivers examined in Japan. Data points are rates estimated in single years. Horizontal and vertical bars are standard errors of GPP and ER, respectively. Thick line and grey area represent the regression line with 95% confidence intervals. Dashed line indicates 1:1 line.

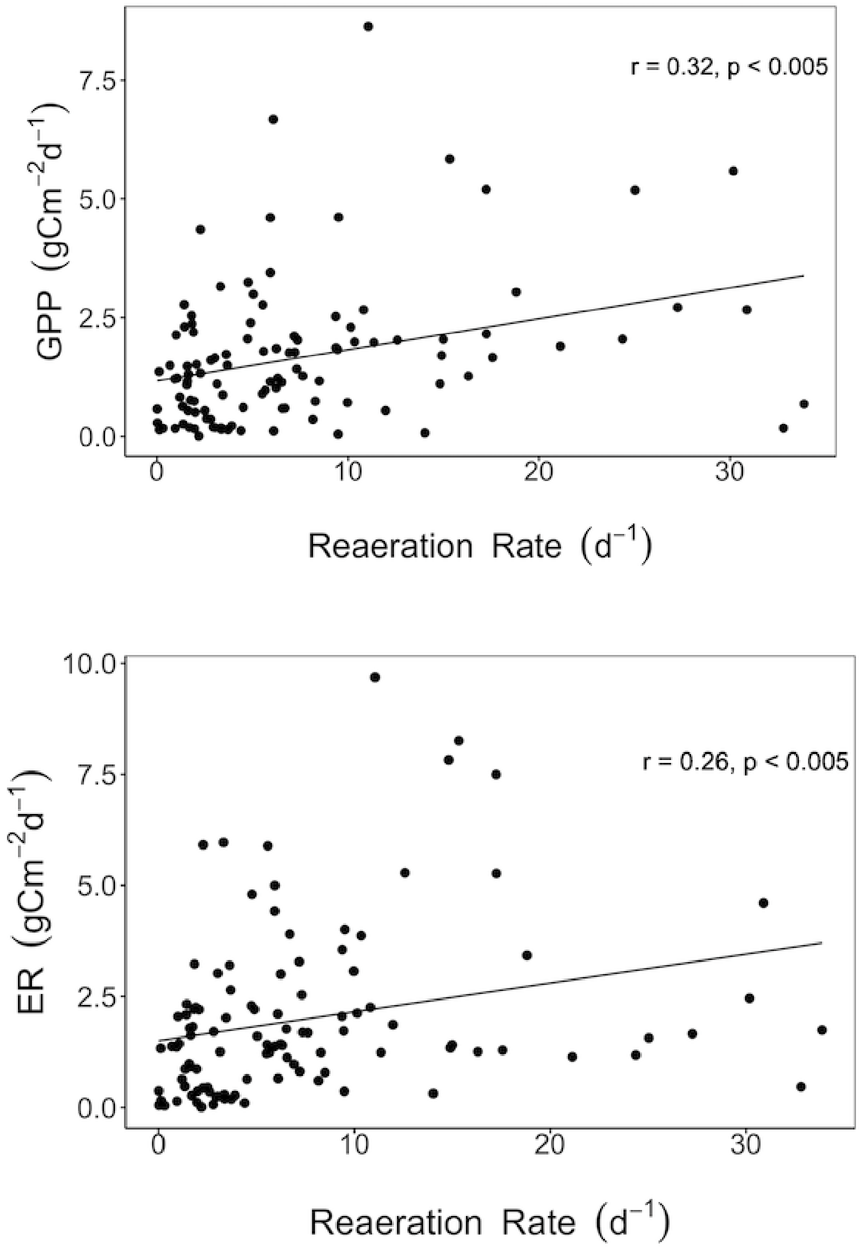

Fig S4: Relationship between GPP and ER, and reaeration rate in the Japanese rivers.

Supplementary Table S1. Geographical positions and details of the observatory sites of the Japanese rivers used in this study.

ID	River	Observatory Site	Latitude	Longitude	Stream Order	Elevation (m)	Depth (m)	Mean water temperature (°C)	Max diel PAR (μ mol m^{-2} s^{-1})	Mean DO (mg O_2/l)
1	Toyohiragawa	Horohirabashi	43.0377	141.3555	5	32	0.62	15.33	472.30	9.93
2	Tokachigawa	Tokachibashi	42.9344	143.2033	6	19	1.02	17.68	748.31	10.58
3	Chitosegawa	Hinodebashi	42.8325	141.6597	4	10	0.15	20.64	577.75	8.61
4	Iwakigawa	Goshogawara	40.8077	140.4375	6	10	1.04	22.01	826.86	7.57
5	Iwakigawa	Kamiiwakibashi	40.5919	140.4169	4	45	0.57	23.32	751.69	8.25
6	Kitakami	Funada bashi	39.8355	141.1613	5	181	0.51	20.94	802.66	8.91
7	Kitakami	Shiwabashi	39.5513	141.1755	6	92	0.96	24.39	730.34	7.98
8	Kitakami	Kanegasaki hashi	39.1966	141.1272	7	41	1.35	25.12	756.36	8.04
9	Mogamigawa	Horinouchi	38.6641	140.2730	6	49	1.44	26.42	801.17	8.33
10	Shinanogawa	Shinanogawa	37.8816	139.0188	7	1	2.33	27.20	877.46	7.76
11	Kujigawa	Sakakibashi	36.4963	140.5544	5	9	0.39	27.68	895.13	7.57
12	Tonegawa	Ashikaga	36.3269	139.4530	5	40	0.26	25.50	842.70	7.82
13	Kisogawa	Kasamatsu	35.3613	136.7569	6	10	0.95	25.25	847.92	7.18
14	Yuragawa	Shimoamadzu	35.3555	135.1152	6	16	0.40	28.72	868.41	7.52
15	Nagara River	Ōyabu ōhashi	35.2966	136.6711	6	10	0.16	25.85	893.90	8.27
16	Shōnaigawa	Biwajima	35.1991	136.8747	5	11	0.48	29.52	807.07	7.28
17	Yahagigawa	Iwatsu	35.0022	137.1666	5	27	0.61	27.01	815.88	7.86
18	Katsuragawa	Miya Maebashi	34.9075	135.7166	6	14	0.41	28.68	787.89	7.34
19	Ujigawa	Miyukibashi	34.8911	135.6994	6	11	1.28	29.70	812.61	6.91
20	Inagawa	Ginbashi	34.8555	135.4155	4	11	0.27	28.82	808.08	8.11
21	Yodogawa	Hirakata Ōhashi	34.8125	135.6316	7	2	1.25	30.73	834.93	6.90
22	Toyokawa	Tō furu/Tougo	34.8105	137.4186	4	9	0.30	27.35	789.05	8.03
23	Ibogawa	Kamikawara	34.8013	134.5630	5	4	0.44	27.91	968.78	7.37
24	Inagawa	Gunkōbashi	34.7988	135.4233	4	12	0.24	27.65	832.25	7.19
25	Kakogawa	Kunikane	34.7975	134.8994	6	13	0.38	28.88	829.35	6.83
26	Kumozugawa	Kumozubashi	34.6466	136.5130	4	6	0.26	28.03	802.07	8.69
27	Yamatogawa	Asaka	34.5858	135.5019	5	6	0.45	29.67	844.73	6.10
28	Miyagawa	Watarai-bashi	34.4891	136.6855	5	10	0.43	26.92	727.88	7.93
29	Chikugogawa	Kurumeōhashi	33.3292	130.5261	7	2	0.65	27.67	871.12	8.92
30	Mimigawa	Yamagehei	32.3862	131.5264	4	19.3	1.2	22.52	857.73	9.20

Table S2. Results of generalized linear mixed model for gross primary production (GPP), ecosystem respiration (ER) and net ecosystem production (NEP) in the Japanese rivers.

Gross primary production rate (GPP) model summary				
Random Variable	Variance	Std.Dev		
Year	0.20	0.44		
Residual	2.12	1.45		
Fixed Variable	Estimate	Std. Error	t-value	P value
(Intercept)	-3.11	2.22	-1.39	0.16
Latitude	0.12	0.06	2.13	0.03
Ecosystem respiration rate (ER) model summary				
Random Variable	Variance	Std.Dev		
Year	0.00	0.00		
Residual	3.47	1.86		
Fixed Variable	Estimate	Std. Error	t-value	P value
(Intercept)	-3.74	2.73	-1.37	0.17
Latitude	0.15	0.07	2.09	0.03
Net ecosystem production rate (NEP) model summary				
Random Variable	Variance	Std.Dev		
Year	0.06	0.24		
Residual	1.77	1.33		
Fixed Variable	Estimate	Std. Error	t-value	P value
(Intercept)	1.53	1.99	0.77	0.44
Latitude	-0.05	0.05	-0.93	0.35

Table S3. Summer GPP and ER of various rivers estimated in previous studies.

Citation	Name of river or stream	Latitude	GPP (g C m^{-2} d^{-1})	ER (g C m^{-2} d^{-1})
Aristegi et al. (2009)	Aitzu	43.026111	10.425	4.425
Aristegi et al. (2009)	Aizarnazabal	43.026111	0.7125	1.0125
Aristegi et al. (2009)	Alegia	43.026111	6.8625	6.45
Aristegi et al. (2009)	Altzola	43.026111	13.2375	2.3625
Aristegi et al. (2009)	Amorebieta	43.026111	4.0875	1.3875
Aristegi et al. (2009)	Balmaseda	43.026111	3.1875	3.825
Aristegi et al. (2009)	Berriatua	43.026111	2.1	1.125
Aristegi et al. (2009)	Elorrio	43.026111	1.5	1.4625
Aristegi et al. (2009)	Erenozu	43.026111	4.2	3.75
Aristegi et al. (2009)	Estanda	43.026111	1.2375	0.4875
Aristegi et al. (2009)	Gardea	43.026111	3.4875	4.725
Aristegi et al. (2009)	Herrerias	43.026111	3.5625	1.3875
Aristegi et al. (2009)	Lasarte	43.026111	3.6375	3.7125
Aristegi et al. (2009)	Leitzaran	43.026111	2.0625	3.3
Aristegi et al. (2009)	Muxika	43.026111	3.225	2.0625
Aristegi et al. (2009)	Oiartzun	43.026111	0.675	0.4125
Aristegi et al. (2009)	Olet	43.026111	5.325	4.0125
Aristegi et al. (2009)	Onati	43.026111	5.775	2.1
Aristegi et al. (2009)	S.Prudentzio	43.026111	4.0875	2.5125
Aristegi et al. (2009)	Sodupe	43.026111	0	3
Benson (2010)	Site 4	64.804217	0.73875	1.575
Benson (2010)	Site 4	64.804217	0.70125	2.46
Benson (2010)	Site 3	64.817533	0.615	1.9125
Benson (2010)	Site 3	64.817533	0.7725	2.68125
Benson (2010)	Site 2	64.880783	0.81	1.89375
Benson (2010)	Site 2	64.880783	0.72	2.43
Benson (2010)	Site 1	64.898483	1.46625	3.34875
Benson (2010)	Site 1	64.898483	0.97125	3.35625
Bernot et al. (2010)	Grande	18.16	1.95	2.85
Bernot et al. (2010)	Maizales	18.23	2.7375	1.9875
Bernot et al. (2010)	Ceiba	18.27	3.4875	4.3875
Bernot et al. (2010)	RIT	18.28	0.1875	1.6875
Bernot et al. (2010)	Bisley	18.32	0.01875	0.9
Bernot et al. (2010)	Pared	18.33	0.15	0.15
Bernot et al. (2010)	Vaca	18.34	1.1625	5.8875
Bernot et al. (2010)	Mtrib	18.37	2.6625	2.775
Bernot et al. (2010)	Petunia	18.39	0.1125	1.725
Bernot et al. (2010)	Sycamore Ck	33.75	1.05	1.4625
Bernot et al. (2010)	Blacks Branch	34.94	0.1875	3.2625
Bernot et al. (2010)	Jerry Branch	34.96	0.1875	1.6875
Bernot et al. (2010)	Mud Creek	34.99	0.0375	2.925
Bernot et al. (2010)	Hugh White Creek	35.05	0.0375	0.825
Bernot et al. (2010)	Cunningham Creek	35.05	0.01875	1.95

Bernot et al. (2010)	Hoglot Branch	35.09	0.1125	0.6
Bernot et al. (2010)	Crawford Branch	35.18	1.125	2.4375
Bernot et al. (2010)	Rio Rancho	35.2	2.4375	3.75
Bernot et al. (2010)	San Pedro	35.21	1.2375	1.9875
Bernot et al. (2010)	Bernalillo drain	35.33	3.3	2.6625
Bernot et al. (2010)	Sugarloaf Creek	35.38	0.0375	6.7125
Bernot et al. (2010)	Kings Creek N4D	39.09	0.675	1.1625
Bernot et al. (2010)	Campus Creek	39.19	0.1125	0.1875
Bernot et al. (2010)	Agnorth	39.21	3	2.85
Bernot et al. (2010)	Natalie Creek	39.23	0.075	0.4125
Bernot et al. (2010)	Arcadia	42.27	0.3	5.2875
Bernot et al. (2010)	Honeysuckle	42.31	0.0375	2.9625
Bernot et al. (2010)	Sawmill Brook	42.52	0.01875	0.45
Bernot et al. (2010)	IS_104	42.54	0.2625	3.4125
Bernot et al. (2010)	Sand Creek	42.58	0.075	0.75
Bernot et al. (2010)	IS_118	42.58	0.0375	1.5
Bernot et al. (2010)	Boxford	42.64	0.01875	5.475
Bernot et al. (2010)	Black Brook	42.64	0.225	1.6875
Bernot et al. (2010)	Runaway Brook	42.65	2.7375	4.125
Bernot et al. (2010)	Long Meadow Brook	42.65	1.4625	3.075
Bernot et al. (2010)	Gravelly Brook	42.66	0.075	4.2375
Bernot et al. (2010)	Wayland	42.67	0.675	1.5375
Bernot et al. (2010)	Steinke Drain	42.71	0.3	0.4875
Bernot et al. (2010)	Dorr	42.73	0.2625	3.2625
Bernot et al. (2010)	Cart Creek	42.77	0.075	1.425
Bernot et al. (2010)	Teton Pines	43.53	1.0125	0.5625
Bernot et al. (2010)	Giltner	43.55	6.075	4.275
Bernot et al. (2010)	Golf	43.57	1.575	3.75
Bernot et al. (2010)	Kimball	43.57	5.1	4.5
Bernot et al. (2010)	Headquarters	43.57	1.2375	2.6625
Bernot et al. (2010)	Ditch	43.66	1.05	1.5
Bernot et al. (2010)	Spread	43.79	1.2	3.675
Bernot et al. (2010)	Two Oceans	43.88	1.0875	4.725
Bernot et al. (2010)	Amazon	44.04	1.05	1.8375
Bernot et al. (2010)	Camp	44.12	0.1125	1.8375
Bernot et al. (2010)	Mack	44.22	0.075	1.8
Bernot et al. (2010)	Potts	44.26	0.1125	5.3625
Bernot et al. (2010)	Courtney	44.36	1.125	1.5
Bernot et al. (2010)	Oak	44.56	0.3	2.5875
Bernot et al. (2010)	Oak	44.57	0.375	0.375
Bernot et al. (2010)	Oak	44.61	0.15	0.375
Bernot et al. (2010)	Periwinkle	44.62	4.425	3.675
Betts and Jones (2009)	C2	65.16	0.5625	1.6875
Betts and Jones (2009)	C4	65.16	0.3375	0.45
Betts and Jones (2009)	P6 burned	65.16	0.9	2.475
Bott et al. (2006)	Buck and Doe run, Meadow	39.921389	1.86375	3.12375

Bott et al. (2006)	Buck and Doe Run, Forest	39.925556	0.55125	1.14375
Bott et al. (2006)	Big Springs, Forest	39.930278	0.2025	1.96125
Bott et al. (2006)	Birch Run, Forest	39.930278	0.2325	2.04375
Bott et al. (2006)	Doe Wister, Forest	39.9025	0.61875	1.845
Bott et al. (2006)	Fishers, Forest	39.928889	0.3825	1.2375
Bott et al. (2006)	Gramies, Forest	39.688333	0.435	0.99375
Bott et al. (2006)	Hannums, Forest	39.899167	0.19125	1.16625
Bott et al. (2006)	Moorheads, Forest	39.880833	0.03375	2.01375
Bott et al. (2006)	Pocopson, Forest	39.903333	0.6675	1.005
Bott et al. (2006)	Teters, Forest	39.874722	0.0975	0.9
Bott et al. (2006)	West Branch WC Cr, Meadow	39.767778	0.435	1.18875
Bott et al. (2006)	Wests, Forest	39.898333	0.0075	1.2375
Bott et al. (2006)	White Clay Cr, Forest	39.863056	0.69375	1.17
Bott et al. (2006)	Big Springs, Meadow	39.931944	0.81	2.79375
Bott et al. (2006)	Birch run, Meadow	39.931944	1.33875	1.605
Bott et al. (2006)	Doe Wister, Meadow	39.903889	1.9875	1.8975
Bott et al. (2006)	Fishers, Meadow	39.927778	0.80625	1.1475
Bott et al. (2006)	Grammies, Meadow	39.684722	1.95	2.235
Bott et al. (2006)	Hannums, Meadow	39.900833	0.6225	2.925
Bott et al. (2006)	Moorheads, Meadow	39.876944	1.275	2.49375
Bott et al. (2006)	Pocopson, Meadow	39.903056	0.975	1.45875
Bott et al. (2006)	Teters, Meadow	39.872222	0.76875	2.8425
Bott et al. (2006)	West Branch WC Cr, Forest	39.7675	0.08625	0.6675
Bott et al. (2006)	Wests, Meadow	39.900556	0.29625	2.43
Bott et al. (2006)	White clay cr, Meadow	39.768889	0.75375	1.0425
Bott et al. (2006)	Kisco	41.196389	0.075	0.3
Bott et al. (2006)	Croton	41.210171	0.45	2.25
Bott et al. (2006)	Cross	41.26	0.3375	1.125
Bott et al. (2006)	Muscoot	41.2694	0.15	0.9375
Bott et al. (2006)	Neversink	41.357222	0.9375	3
Bott et al. (2006)	Rondout	41.92	1.3125	1.5
Bott et al. (2006)	Esopus	42.015556	1.4625	3
Bott et al. (2006)	Bushkill	42.14746	1.575	3
Bott et al. (2006)	West Branch Deleware	42.453611	1.2	1.875
Bott et al. (2006)	Schoarie	42.941111	0.7875	1.5
Cappelletti (2006)	Kuparuk River, Ref	68.633333	0.5625	6.75
Cappelletti (2006)	Kuparuk River, Fertilized	68.633333	1.05	7.125
Chen (2013)	WM	43.052222	4.371225	4.62435
Chen (2013)	GM	43.277228	4.7625	4.22625
Chen (2013)	BL	43.386056	4.80375	9.346875
Chen (2013)	BL	43.386056	6.7575	11.2875
Chen (2013)	BL	43.386056	6.429375	8.709375
Chen (2013)	BL	43.386056	3.594375	7.119375
Chen (2013)	BP	43.481861	2.61	2.5790625
Chen (2013)	BP	43.481861	2.9775	2.13375
Chen (2013)	BP	43.481861	3.009375	2.3175

Chen (2013)	SPb	43.484236	4.0425	4.0575
Chen (2013)	SPb	43.484236	3.4125	3.4125
Chen (2013)	Spa	43.534567	0.25875	0.3375
Chen (2013)	Spa	43.534567	0.1875	0.1875
Chen (2013)	5F	43.640064	1.6725	2.64375
Chen (2013)	5F	43.640064	0.8025	1.23
Chen (2013)	5NF	43.666628	3.345	3.414375
Chen (2013)	3NF	43.699914	1.2525	2.4825
Chen (2013)	3NF	43.699914	1.2	2.94375
Chen (2013)	4F	43.705731	0.691875	1.505625
Chen (2013)	4F	43.705731	1.125	2.38875
Chen (2013)	4NF	43.707889	0.84375	1.2675
Chen (2013)	4NF	43.707889	1.15875	1.935
Chen (2013)	2NF	43.714703	0.271875	0.999375
Chen (2013)	2NF	43.714703	0.47625	2.10375
Chen (2013)	3F	43.7289	1.44375	2.229375
Chen (2013)	3F	43.7289	0.73875	1.27125
Chen (2013)	2F	43.734567	0.354375	1.400625
Chen (2013)	2F	43.734567	0.34875	0.9375
Davis (2012)	South Fork Humboldt	40.666667	5.875125	4.024875
Demars et al. (2016)	PAR 19	52.823583	2.5425	7.5
Demars et al. (2016)	PAR 21	52.82375	1.02375	7.875
Demars et al. (2016)	PAR 10	52.824361	1.92	3.825
Demars et al. (2016)	PAR 14	52.8245	0.75	2.1375
Demars et al. (2016)	TOR 2	63.933389	0.09375	0.15
Demars et al. (2016)	TOR 1	63.933444	1.3875	2.4
Demars et al. (2016)	TOR 3	63.934389	4.7175	4.425
Demars et al. (2016)	TOR 4	63.935194	0.77625	2.1
Demars et al. (2016)	TOR 7	63.954556	3.04875	4.575
Demars et al. (2016)	TOR 6	63.955028	0.53625	0.5625
Demars et al. (2016)	HG 18	64.009833	5.67375	6.3
Demars et al. (2016)	HG 19	64.010306	9.38625	13.05
Demars et al. (2016)	HG 20	64.010833	13.32	17.325
Demars et al. (2016)	HG 21	64.011056	16.37625	19.2
Demars et al. (2016)	HG 37	64.012222	3.2325	6.375
Demars et al. (2016)	HG 27	64.012806	1.6425	1.5
Demars et al. (2016)	HG 22	64.018167	5.26125	4.05
Demars et al. (2016)	HG 23	64.018167	3.69	1.35
Demars et al. (2016)	HG 24	64.019056	4.9125	3.75
Demars et al. (2016)	HG 35	64.025583	6.405	15.9375
Demars et al. (2016)	HG 36	64.02625	8.22	6.4875
Demars et al. (2011)	1	64.05	7.6125	10.575
Demars et al. (2016)	HG 25	64.059389	0.7875	1.3875
Demars et al. (2016)	HG 26	64.060111	6.675	16.0875
Demars et al. (2016)	HEN 12	64.080028	1.575	4.275
Demars et al. (2016)	HEN 1	64.089944	7.63125	10.575

Demars et al. (2016)	HEN 5	64.092694	10.35	14.25
Demars et al. (2016)	HEN 2	64.093	5.3475	7.05
Demars et al. (2016)	HEN 3	64.093917	1.85625	6.4125
Demars et al. (2016)	HEN 4	64.094278	0.84375	0.9375
Demars et al. (2016)	HEN 6	64.094472	6.3	6.8625
Demars et al. (2016)	HEN 7	64.095639	1.6575	2.625
Demars et al. (2016)	HEN 8	64.09575	5.1075	25.05
Demars et al. (2016)	HEN 9	64.096361	5.89125	9.5625
Demars et al. (2016)	HEN 10	64.097194	3.91125	8.9625
Demars et al. (2016)	HEN 11	64.098028	3.465	3.6375
Demars et al. (2016)	HEN 14	64.100528	0.82125	1.5375
Demars et al. (2011)	14	64.517778	0.825	1.5375
Demars et al. (2016)	KER 31	64.64575	0.4875	1.1625
Demars et al. (2016)	VON 4	64.679444	0.94125	0.9375
Demars et al. (2016)	VON 5	64.684083	0.19875	0.825
Demars et al. (2016)	VON 6	64.686056	0.62625	1.2
Demars et al. (2016)	VON 7	64.687667	3.3375	3.3375
Demars et al. (2016)	KER 43	64.688167	0.7275	8.2125
Demars et al. (2016)	KER 42	64.689472	0.4125	2.6625
Demars et al. (2016)	VON 1	64.6895	3.075	3.6375
Demars et al. (2016)	VON 8	64.689528	0.3825	0.15
Demars et al. (2016)	KER 41	64.689556	1.035	3.825
Demars et al. (2016)	VON 2	64.690694	1.11	2.1
Demars et al. (2016)	KER 40	64.692361	2.51625	20.2875
Demars et al. (2016)	KVE 50	64.865778	1.53375	1.8375
Demars et al. (2016)	KVE 51	64.865861	0.6525	1.5375
Demars et al. (2011)	5	73.368056	10.35	14.25
Demars et al. (2011)	2	73.66	5.3625	7.05
Demars et al. (2011)	3	74.58	1.8375	6.4125
Demars et al. (2011)	4	74.9347	0.825	0.9375
Demars et al. (2011)	6	75.151389	6.3	6.8625
Demars et al. (2011)	7	76.301389	1.65	2.625
Demars et al. (2011)	8	76.418056	5.1	25.05
Demars et al. (2011)	9	77.03472	5.8875	9.5625
Demars et al. (2011)	12	77.351111	1.575	4.275
Demars et al. (2011)	10	78.684722	3.9	8.9625
Demars et al. (2011)	11	78.684722	3.45	3.6375
Dodds et al. (2018)	Clarks	39.000556	0.96375	0.77625
Dodds et al. (2018)	Saline	39.003889	1.05375	1.0875
Dodds et al. (2018)	Paxico	39.064722	0.75375	0.72
Dodds et al. (2018)	Salt	39.139167	1.6875	1.83
Dodds et al. (2018)	Rock	39.264722	0.4275	0.58875
Dodds et al. (2018)	Stranger	39.447778	0.2025	0.47625
Dodds et al. (2018)	Grasshopper	39.584444	0.405	0.73875
Dodds et al. (2018)	Muddy	39.628611	1.12125	1.18125
Dodds et al. (2018)	Republican	39.984167	0.79875	0.8475

Dodds et al. (2018)	Delaware	40.403889	0.135	0.16125
Duffer and Dorris. (1966)	Blue River Reach 12-13	33.996944	1.12875	2.87625
Duffer and Dorris. (1966)	Blue River Reach 5-6	33.996944	2.56875	4.11
Duffer and Dorris. (1966)	Blue River Reach 6-7	33.996944	8.01375	4.725
Escoffer et al. (2016)	Siene River	49.434639	1.425	1.63125
Fellows et al. (2006)	Hugh White Cr	35.0597	0.01875	0.37125
Fellows et al. (2006)	East Fork Walker Branch	35.966667	0.045	1.42875
Fellows et al. (2001)	Gallina Cr	36.0378	0.6375	5.5125
Fellows et al. (2001)	Gallina Cr	36.0378	0.075	2.5125
Fellows et al. (2006)	Gallina Cr	36.0378	0.525	1.13625
Fellows et al. (2001)	Rio Calaveras	38.197222	0.225	0.8625
Fellows et al. (2001)	Rio Calaveras	38.197222	0.1875	1.0875
Fellows et al. (2006)	Rio Calaveras	38.197222	0.1725	0.2925
Flemer (1970)	Raritan River, Station I	40.555556	1.7625	1.6875
Hall and Tank, (2013)	Bailey Cr	43.7904	0.39	0.7575
Hall and Tank (2003)	Ditch Cr	43.7904	0.7275	2.41875
Hall and Tank (2003)	Glade Cr trib	43.7904	0.405	4.9875
Hall and Tank (2003)	Lizard Cr	43.7904	0.225	1.5375
Hall and Tank (2003)	Moose-Wilson rd Cr	43.7904	0.0675	2.26875
Hall and Tank (2003)	North Moran Bay Cr	43.7904	0.13125	2.16
Hall and Tank (2003)	Paintbrush Canyon Cr	43.7904	0.11625	0.70125
Hall and Tank (2003)	Pilgrim Creek	43.7904	0.09	0.36375
Hall and Tank (2003)	Pilgrim Creek	43.7904	0.04875	0.59625
Hall and Tank (2003)	Spread Cr	43.7904	1.16625	3.13875
Hall and Tank (2003)	Two Ocean lake outlet	43.7904	0.61125	3.28875
Hall et al. (2003)	Polecat Creek	44.1044	4.4625	3.8625
Hall et al. (2003)	Polecat Creek	44.1044	3.7125	4.81875
Hart (2013)	Balls Creek	35.68544	0.1099125	0.699657534
Hart (2013)	Bates Creek	35.68544	0.1304625	0.219863014
Hart (2013)	Caler Main	35.68544	0.3675	1.276027397
Hart (2013)	Cowee Creek	35.68544	0.3040875	0.811643836
Hart (2013)	Crawford Branch	35.68544	0.3873	1.202054795
Hart (2013)	Jones Creek	35.68544	0.334931507	0.878424658
Hart (2013)	Ray Branch	35.68544	0.230136986	1.293493151
Hart (2013)	Skeenah Creek	35.68544	0.546575342	1.159931507
Hart (2013)	Watauga Creek	35.68544	0.25890411	0.714041096
Holtgrieve (2011)	Pick Creek	59.55	0.6499875	2.7
Holtgrieve (2011)	Pick Creek	59.55	0.75	0.975
Holtgrieve (2011)	Seventh Creek	59.581	0.45	0.61875
Holtgrieve (2011)	Berm Creek	59.596	0.225	0.21225
Iwata et al. (2007)	Ai S.	35.666667	1.5975	1.5525
Iwata et al. (2007)	Ani S.	35.666667	2.4	6.34125
Iwata et al. (2007)	Byodo S.	35.666667	2.89125	5.35125
Iwata et al. (2007)	Fuji S.	35.666667	0.97125	2.5125
Iwata et al. (2007)	Hi R.	35.666667	0.73875	3.63225
Iwata et al. (2007)	Higashihanawa S.	35.666667	2.42625	4.28625

Iwata et al. (2007)	Hirusawa S. I	35.666667	2.19	3.6
Iwata et al. (2007)	Hirusawa S. II	35.666667	1.45875	4.24125
Iwata et al. (2007)	Kamata S. I	35.666667	0.7125	2.50875
Iwata et al. (2007)	Kamata S. II	35.666667	4.49625	5.76375
Iwata et al. (2007)	Kurosawa S.	35.666667	4.4475	5.49
Iwata et al. (2007)	Nagare S.	35.666667	1.69875	3.12375
Iwata et al. (2007)	Nigori S.	35.666667	0.43875	5.81625
Iwata et al. (2007)	Nishi S.	35.666667	4.39125	6.86625
Iwata et al. (2007)	Omo R. II	35.666667	2.17125	4.93875
Iwata et al. (2007)	Omo R. I	35.666667	1.43625	3.42375
Iwata et al. (2007)	Otouto S.	35.666667	3.24	7.56
Iwata et al. (2007)	Sanno S.	35.666667	2.11125	4.08375
Iwata et al. (2007)	Shimoda S.	35.666667	1.84875	3.68625
Iwata et al. (2007)	Shinmei S. I	35.666667	8.32875	12.5625
Iwata et al. (2007)	Shinmei S. II	35.666667	2.5275	3.815625
Iwata et al. (2007)	Takisawa S.	35.666667	4.69875	8.925
Iwata et al. (2007)	Yoko S.	35.666667	1.95	4.99125
Kaenel et al. (2000)	Muhlibach	47.676	4.6875	3.3375
Kupilas et al. (2017)	D	51.44093	2.934375	4.215
Kupilas et al. (2017)	R1	51.44093	2.754375	3.38625
Kupilas et al. (2017)	R2	51.44093	3.984375	5.74125
Naegeli and Uehlinger (1997)	Necker River	47.384	2.8125	2.5875
Naegeli and Uehlinger (1997)	Necker River	47.384	3	2.625
Naegeli and Uehlinger (1997)	Necker River	47.384	2.6625	2.4375
Naegeli and Uehlinger (1997)	Necker River	47.384	1.275	1.8375
Naegeli and Uehlinger (1997)	Necker River	47.384	2.2125	2.1
Naegeli and Uehlinger (1997)	Necker River	47.384	2.3625	2.2875
Naegeli and Uehlinger (1997)	Necker River	47.384	1.0125	1.7625
Naegeli and Uehlinger (1997)	Necker River	47.384	1.5375	1.95
Naegeli and Uehlinger (1997)	Necker River	47.384	0.675	1.725
Rasmussen (2011)	s1	64.05	4.875	9.5625
Rasmussen (2011)	s2	64.05	3.6	5.5125
Rasmussen (2011)	s3	64.05	0.9	3.525
Rasmussen (2011)	s4	64.05	0.2475	2.1
Rovelli et al. (2017)	GN	51.042947	0.372	0.47925
Rovelli et al. (2017)	CW	51.156453	0.694125	0.62925
Song et al. (2018)	E	44.222825	0.0555625	0.3480125
Song et al. (2018)	E	68.647897	0.550817647	1.198228676
Song et al. (2018)	E	68.647897	0.6678375	0.834075
Song et al. (2018)	E	68.648258	0.256579412	0.546511765
Song et al. (2018)	E	68.643646	0.38269	1.2509875
Song et al. (2018)	E	68.643646	0.241096875	0.44240625
Song et al. (2018)	E	68.646108	0.334885	1.01712
Song et al. (2018)	E	68.636796	0.02082	0.126075
Song et al. (2018)	E	68.636796	0.02805	0.2541
Song et al. (2018)	E	68.63687	0.01134375	0.11064375

Song et al. (2018)	S		68.633222	0.4686	0.8328
Song et al. (2018)	S		68.633222	0.777820833	1.599479167
Song et al. (2018)	S		68.642999	0.2673	0.380325
Song et al. (2018)	S		68.634146	0.0672	0.1668
Song et al. (2018)	S		68.634146	1.2075	4.689
Song et al. (2018)	E		39.10006944	0.295907143	0.422292857
Song et al. (2018)	E		39.10043889	0.11985	0.19003125
Song et al. (2018)	S		39.10205	0.4438125	1.3091625
Song et al. (2018)	E		39.10043889	0.0831375	0.11745
Song et al. (2018)	S		39.105786	0.03765	0.173775

References

1. Aristegi, L., O. Izagirre, and A. Elosegi. 2010. Metabolism of Basque streams measured with incubation chambers. Limnetica 29: 301–310.
2. Benson, E. R. 2010. Relationships between ecosystem metabolism, benthic macroinvertebrate desities, and envirnmental variables in a sub-arctic Alaskan River. University of Alaska Fairbanks.
3. Bernot, M. J., D. J. Sobota, R. O. Hall, and others. 2010. Inter-regional comparison of land-use effects on stream metabolism. Freshw. Biol. 55: 1874–1890. doi:10.1111/j.1365-2427.2010.02422.x
4. Betts, E. F., and J. B. Jones. 2009. Impact of Wildfire on Stream Nutrient Chemistry and Ecosystem Metabolism in Boreal Forest Catchments of Interior Alaska. Arctic, Antarct. Alp. Res. 41: 407–417. doi:10.1657/1938-4246-41.4.407
5. Bott, T. L., J. D. Newbold, and D. B. Arscott. 2006. Ecosystem metabolism in piedmont streams: Reach geomorphology modulates the influence of riparian vegetation. Ecosystems 9: 398–421. doi:10.1007/s10021-005-0086-6
6. Cappelletti, C. 2006. Photosynthesis and Respiration in an arctic Tundra River : Modification and Application of the Wholestream metabolism method and the influence of physical, biological, and chemical variables.
7. Chen, G. 2013. Ecosystem oxygen metabolism in an impacted temperate river network: Application of the δ18O-DO approach.
8. Davies, C. J., C. H. Fritsen, E. D. Wirthlin, and J. C. Memmott. 2012. High rates of primary productivity in a semi-arid tailwater: Implications for self-regulated production. River Res. Appl. 21: 1820–1829. doi:10.1002/rra
9. Demars, B. O. L., G. M. Gíslason, J. S. Ólafsson, J. R. Manson, N. Friberg, J. M. Hood, J. J. D. Thompson, and T. E. Freitag. 2016. Impact of warming on CO2emissions from streams countered by aquatic photosynthesis. Nat. Geosci. 9: 758–761. doi:10.1038/ngeo2807
10. Demars, B. O. L., J. Russell Manson, J. S. Ólafsson, and others. 2011. Temperature and the metabolic balance of streams. Freshw. Biol. 56: 1106–1121. doi:10.1111/j.1365-2427.2010.02554.x
11. Dodds, W. K., S. A. Higgs, M. J. Spangler, and others. 2018. Spatial heterogeneity and controls of ecosystem metabolism in a Great Plains river network. Hydrobiologia 813: 85–102. doi:10.1007/s10750-018-3516-0

12. Duffer, W. R., and T. C. Dorris. 1966. Primary Productivity in a Southern Great Plains Stream.
13. Escoffier, N., N. Bensoussan, L. Vilmin, N. Flipo, V. Rocher, A. David, F. Métivier, and A. Groleau. 2016. Estimating ecosystem metabolism from continuous multi-sensor measurements in the Seine River. Environ. Sci. Pollut. Res. 1–17. doi:10.1007/s11356-016-7096-0
14. Fellows, C. S., H. M. Valett, C. N. Dahm, and S. Fellows. 2001. Whole-stream metabolism in two montane streams: Contribution of the hyporheic zone. Limol.Oceanogr 46: 523–531.
15. Fellows, C. S., H. M. Valett, C. N. Dahm, P. J. Mulholland, and S. A. Thomas. 2006. Coupling nutrient uptake and energy flow in headwater streams. Ecosystems 9: 788–804. doi:10.1007/s10021-006-0005-5
16. Flemer, D. A. 1970. Primary productivity of the North Branch of the Raritan River, New Jersey. Hydrobiologia 35: 273–296. doi:10.1007/BF00181732
17. Hall R. O., J., J. L. Tank, and M. F. Dybdahl. 2003. Exotic snails dominate nitrogen and carbon cycling in a highly productive stream. Frontiers in Ecology and the Environment 1(8):407--411. 2.
18. Hall, R. O., and J. L. Tank. 2003. Ecosystem metabolism controls nitrogen uptake in streams in Grand Teton National Park, Wyoming. Limnol. Oceanogr. 48: 1120–1128. doi:10.4319/lo.2003.48.3.1120
19. Hart, A. M. 2013. Seasonal Variation in Whole Stream Metabolism across Varying Land Use Types Adam Michael Hart Thesis submitted to the faculty of the Virginia Polytechnic Institute and State University in partial fulfillment of the requirements for the degree of Master of. 66.
20. Holtgrieve, G. W., and D. E. Schindler. 2011. Marine-derived nutrients, bioturbation, and ecosystem metabolism: Reconsidering the role of salmon in streams. Ecology 92: 373–385. doi:10.1890/09-1694.1
21. Iwata, T., T. Takahashi, F. Kazama, and others. 2007. Metabolic balance of streams draining urban and agricultural watersheds in central Japan. Limnology 8: 243–250. doi:10.1007/s10201-007-0212-6
22. Kaenel, B. R., H. Buehrer, and U. Uehlinger. 2000. Effects of aquatic plant management on stream metabolism and oxygen balance streams. Freshw. Biol. 45: 85–95. doi:10.1046/j.1365-2427.2000.00618.x

23. Kupilas, B., D. Hering, A. Lorenz, C. Knuth, and B. Gucker. 2017. Hydromorphological restoration stimulates river ecosystem metabolism. Biogeosciences 14: 1989–2002. doi:10.5194/bg-14-1989-2017
24. Naegeli, M. W., and U. Uehlinger. 1997. Contribution of the hyporheic zone to ecosystem metabolism in a prealpine gravel-bed river. J. North Am. Benthol. Soc. 16: 794–804.
25. Rasmussen, J. J., A. Baattrup-Pedersen, T. Riis, and N. Friberg. 2011. Stream ecosystem properties and processes along a temperature gradient. Aquat. Ecol. 45: 231–242. doi:10.1007/s10452-010-9349-1
26. Rovelli, L., K. M. Attard, A. Binley, C. M. Heppell, H. Stahl, M. Trimmer, and R. N. Glud. 2017. Reach-scale river metabolism across contrasting sub-catchment geologies: Effect of light and hydrology. Limnol. Oceanogr. 62: S381–S399. doi:10.1002/lno.10619
27. Song, C., W. K. Dodds, J. Rüegg, and others. 2018. Continental-scale decrease in net primary productivity in streams due to climate warming. Nat. Geosci. 11. doi:10.1038/s41561-018-0125-5
